# A 10-Year-Old Girl’s Dysfunctional ‘Self-Help’ in ADHD: Suppression of Hyperkinetic Symptoms via Self-Induced Weight Loss in the Context of Anorexia Nervosa—A Case Report

**DOI:** 10.3390/children10091509

**Published:** 2023-09-05

**Authors:** Stefan Mestermann, Valeska Stonawski, Lea Böhm, Oliver Kratz, Gunther H. Moll, Stefanie Horndasch

**Affiliations:** Department of Child and Adolescent Mental Health, University Hospital Erlangen, Friedrich-Alexander-Universität Erlangen-Nürnberg (FAU), 91054 Erlangen, Germanystefanie.horndasch@uk-erlangen.de (S.H.)

**Keywords:** child and adolescent psychiatry, mental health, ADHD, anorexia nervosa, comorbidity, weight loss, methylphenidate

## Abstract

Anorexia Nervosa (AN) and Attention Deficit Hyperactivity Disorder (ADHD) are frequent mental disorders in child and adolescent psychiatry. Comorbidity of these disorders is, however, rare among minors. Thus, little is known about their mutual impact on illness development as well as diagnostic and therapeutic influencing factors. We report the case of a 10-year old girl with AN and massive underweight. At the age of 5, ADHD had been diagnosed. Application of ADHD-specific medication had been refused by her caregiver. As of 3rd grade, hyperkinetic symptoms were significantly reduced, which was later linked to beginning AN-induced weight loss. At inpatient admission, no clinically relevant ADHD-related symptoms were present. Accompanying weight gain, rather ‘sudden’ appearance of attention difficulties, motoric hyperactivity and impulsivity were reported, widely impairing our patient’s schoolwork and further daily life. Methylphenidate medication showed good clinical response and tolerability. We hypothesize that the former massive underweight had suppressed ADHD-specific behaviour. AN with significant weight loss could possibly mask hyperkinetic symptoms in children. Thus, sufficient clinical diagnostics and intense monitoring during ED treatment are required. Physicians and therapists should be sensitized for interactions in the joint occurrence of these mental disorders among minors.

## 1. Introduction

In child and adolescent psychiatric healthcare, AN and ADHD are frequently treated disorders. In the past few years, rising numbers and higher symptom severity of EDs, particularly AN, were reported in the international literature, not least as a consequence of the COVID-19-pandemic. In Germany, the prevalence of eating disorders among female adolescents has increased by 54% (9.8/1000) from 2019 to 2021 [1]. AN onset among minors is peaking in adolescence, around age 14–17, with a tendency towards higher numbers of early onsets before the age of 14 [2]. AN is a severe psychiatric disease and shows the highest mortality of all psychiatric disorders. It is a leading cause of death among female minors [3].

In addition to multiple somatic complications (e.g., endocrine impairment presenting as amenorrhoea in females), formal thought disorder (FTD) with rumination on eating, weight and body image as well as body image disturbance are among the psychiatric symptoms of AN. AN-specific behaviour includes self-imposed diets, excessive physical activity and possibly vomiting or purging (e.g., via abuse of laxatives or diuretics) [4].

ADHD is a neurodevelopmental disorder characterized by the main symptoms of inattention, motoric hyperactivity and impulsivity. Worldwide prevalence of ADHD is around 5%, peaking at childhood age [5] and with higher rate among boys compared to girls [6]. DSM-V defines onset age <12 y as diagnostic criterion [7]. There are no reports about rising incidence of ADHD related to the COVID-19 pandemic. However, the literature reports the worsening of pre-existing symptoms in ADHD patients [8].

Comorbidity of ADHD and AN is, however, very rare in clinical cases of younger children, particularly in females. Thus, little is known about mutual pathogenesis, impact on illness development or diagnostic and therapeutic influencing factors. Literature links ADHD mostly to EDs with impulsive behaviour, like Bulimia Nervosa (BN), Binge Eating Disorder (BED), AN with self-induced vomiting and obesity among youths or adults [9,10]. ‘Picky eating’ and other EDs like avoidant restrictive food intake disorder (ARFID) are also more commonly comorbid to ADHD [11]. Data on AN and ADHD comorbidity are scarce, but an AN prevalence of 2.4% among females with ADHD has been reported [12]. As underweight following AN is known to induce multiple somatic, psychiatric and behavioural side effects, an impact of AN on hyperkinetic ADHD symptomatology is probable.

The objective of this article is to present the clinical case of a 10-year old girl with previously diagnosed ADHD who developed AN with weight loss and thereby suppressed her hyperkinetic disorder symptoms. We intend to outline the effects of underweight on ADHD-specific behaviour and its change throughout weight regain during child and adolescent psychiatric therapy.

## 2. Case Presentation

### 2.1. Clinical Presentation

A 10–11-year-old female patient was presented at our Department for Child and Adolescent Mental Health at University Hospital of Erlangen, Germany. Previously, she had undergone paediatric hospital treatment after her parents and day caregivers at school had recognized growing food and fluid refusal. They reported a self-induced weight loss of 7 kg during the past 3 months via selective and restrictive eating. She was admitted to hospital at 27 kg, 1st BMI age percentile [13]. The patient reported AN-specific ideations like the intention to be ‘as thin as possible’, fear of weight-gain, calorie counting and body image disturbance, present for about one year. She stated social media as having a strong influence on her body image and described permanent mental occupation with food and weight. She performed excessive physical activity via gymnastics and workouts for up to 2 h per day. Vomiting and drug abuse were denied. Puberty development was delayed, menarche had not yet occurred. Somatic reasons for weight loss were excluded via clinical examination, blood diagnostics (i.e., hyperthyreosis, celiac disease, inflammatory or malignant diseases) and cranial magnetic resonance imaging (MRI). MRI showed no underlying central nervous pathology. Assessment via the Eating-disorder-inventory (EDI-2) [14] showed values above the cut-off for the subscales ‘drive for thinness’ and ‘interoceptive awareness’. Active-type AN (ICD-10: F50.01) was diagnosed according to ICD-10 criteria following inpatient admission at our ED-specific ward. She suffered from somatic effects of undernutrition like xerosis cutis, brittle hair/nails and constipation, for which she received topic skin care and laxatives (Lactulose, later Macrogol). Calcium and Vitamin D3 substitution 600 mg/400 I.E. was administered for the prevention of osteoporosis. ECG and echocardiography revealed no cardiac pathologies apart from AN-specific bradycardia (heart rate as low as 49/min). A hydrogen breath test revealed lactose malabsorption, wherefore she received a lactose-free nutrition.

Additionally, depressive symptoms like low and dysphoric mood, avolition and anhedonia were present. Her mother reported an affective lability with frequent crying and anger as well as permanent feelings of guilt and insufficiency in the girl. Previously to hospital treatment, the girl had socially withdrawn from peer contacts. She further reported sleep disturbances with frequent night awakenings. She showed anxiety symptoms like social phobia, performance and separation anxiety at admission, which had developed since the beginning of weight loss. The girl suffered from recurring states of high tension particularly connected to eating and tube feeding situations. In this context, she had repeatedly injured herself deliberately via slamming her head against walls or objects.

At the age of 5, ADHD (combined presentation) had been diagnosed due to inattention, hyperactivity and impulsivity. The girl had suffered immensely from low performance at school. She was avoided by peers and repeatedly involved in conflicts with peers and adults, particularly due to her impulsive behaviour. Criteria of oppositional defiant disorder were not met. Administration of stimulant medication had previously been recommended yet refused by her caregivers. The patient had received ergotherapy, motopedic and occupational therapy during early childhood. After attending special school for first two classes, the change to regular elementary school was followed by improvement and ‘disappearance’ of hyperkinetic symptoms, later being linked to the beginning of AN-specific weight loss. This development was also verified by evaluating the girl’s school reports, where improvement of her grades had been noticed. At inpatient admission, no clinically relevant ADHD-related symptoms were present at all. The German questionnaires ‘Diagnosesystem für psychische Störungen nach ICD-10 und DSM-5 für Kinder und Jugendliche’ (DISYPS-III) [15] yielded mostly percentile ranks below the cut-off for ADHD symptoms (Table 1). Family history for psychiatric disorders was positive (depression, eating disorders, schizophrenia, dissocial tendencies, suspected ADHD). Thus, family self-management and structuring was impaired.

### 2.2. Therapeutic Process

Our patient received a highly structured AN-specific cognitive-behavioural treatment program with 3 main and 2 to 3 snack meals per day in an inpatient setting. The girl and her caregivers signed a therapy agreement, through which a target weight (‘minimum weight’) at the 25th age BMI-percentile (35 kg) was defined. Body weight was measured every morning, 3 times per week during later treatment stages. We addressed eating behaviour via contingency management and token economy. Portion sizes were adapted to a weekly checked average weight-gain progress. Due to initially constant rejection of any food or drink intake, feeding via nasogastric tube was required for 3 weeks. After that, food alimentation was initially possible only via sip feeds, before our patient started gradually tolerating oral nutrition. The refeeding process was accompanied by AN-specific psychotherapeutic sessions in individual and group settings several times per week. These sessions comprised, among others, psychoeducation and cognitive restructuring, as well as training of social and emotional skills and body image interventions. During residential and later day-care treatment at our ED-specific ward, our patient was able to gain weight up to her target weight and flexibilize her eating structure. Her mood and anxiety symptoms subsided. She became more open and was able to build up social contacts with peers and adults under constant weight gain. States of high tension decreased and vanished completely. There was no deliberate self-injury during our treatment. Her heart rate normalized, digestion restored under gradual reduction of the laxative dose and her hair and nails revitalized. Our patient attended daily lessons at our Hospital School during her treatment.

Accompanying weight gain, particularly with the 10th age BMI-percentile (31 kg) after about 1.5 months of treatment, the healthcare team, teachers and caregivers reported rather ‘sudden’ clinical (re-)appearance of constant attention difficulties, motoric hyperactivity and impulsivity. The girl was increasingly distractible and unable to pursue executive tasks, at which she had shown good endurance for a longer time span during the first weeks of treatment (e.g., school work, therapeutic interviews or group conversations). Careless mistakes were becoming more frequent. She increasingly showed disruptive behaviour during lessons at the Hospital School by interrupting her teachers or shouting out loudly during class. She was increasingly unable to sit and stand still, stood up frequently during lessons and moved around the classroom. Further, she was almost run over by a car when following a cat onto the streets. This had to be differentiated from the urge to move in order to lose weight, which is typical for AN. As our patient was also significantly less anxious about increasing body weight, this was interpreted as a hyperkinetic symptom. ADHD-specific clinical ratings showed significant increases in values (Table 1). Symptom checklists by the healthcare team and teachers demonstrated maximum ratings for hyperkinetic behaviour after weight gain. Thus, the girl’s schoolwork and daily life were widely impaired by her re-appeared ADHD symptoms. There was also a change in her eating behaviour, which no longer presented restrictive, but increasingly impulsive. Her table manners worsened, in the sense that she spilled parts of her food on herself and the floor because of her motoric hyperactivity and higher talkativeness.

With considerate parental informed consent and psychoeducation particularly regarding the girl’s psychological distress, her caregivers agreed to ADHD-specific medication. Methylphenidate was begun and showed good clinical response and tolerability. After titrating medication up to Medikinet^®^ retard (dosage: 30–0–0–0 mg/d), hyperkinetic symptoms significantly reduced (Table 1). Our patient’s work and social behaviour at school as well as her daily competence among family and peers improved notably. Her eating behaviour became more adequate. She began to attend her regular elementary school class again. The girl was discharged from inpatient treatment after 3 months and continued day-care therapy for another 2 months. However, ADHD symptoms were still present at discharge on a reduced level (Table 1). During day-care therapy, eating structures at home were not kept up similarly to the inpatient setting, so the girl again lost some weight but stayed within the normal weight range without showing significant ED symptoms. In order to maintain daily structures with risk of AN relapse, her caregiver received AN-specific family interventions. Further, youth welfare services provided an outpatient social work family assistance following discharge.

## 3. Discussion

To our knowledge, this is the first clinical case study linking ADHD to AN in a young patient at the age of 10 years. Although increased risks of ADHD–AN comorbidity have been previously demonstrated [16], little is known about mutual pathogenesis, impacts on illness progression or diagnostic and therapeutic influencing factors, particularly among minors. ADHD is more commonly linked to impulse-behaviour EDs, like BN, BED or AN with self-induced vomiting. Our report aimed to highlight a case with a strong effect of AN-induced underweight on hyperkinetic symptoms and their changing throughout weight regain. Physicians and therapists in child and adolescence psychiatry diagnostics should be sensitized for interaction in the joint occurrence of these mental disorders among children.

As behavioural observations and ratings suggest, re-appearance of clinically highly relevant ADHD symptoms was induced by reaching normal weight (10th BMI age percentile). We hypothesize that former massive underweight had suppressed (‘numbed’) ADHD-specific behaviour through as-yet-unknown neurocognitive mechanisms.

Possible appetite-suppressing and thus weight-reducing adverse side effects of stimulant medication have to be considered [17]. Because of the high burden of suffering, we recommended MPH despite these risks, which were carefully monitored and not experienced by our young patient. Contrary to our report, ADHD of minors and adults is typically associated with obesity rather than underweight [18]. Our findings do not match postulations of the activity-based anorexia animal (ABA-) model, that hyperkinetic symptoms are induced by food restriction in rats [19]. In the case of our patient, however, ADHD was present before she developed AN.

Pathophysiologic consequences of underweight include central nervous atrophy and thus neurocognitive impairment. Underlying somatic pathologies, e.g., cerebral malignancy, should be ruled out, particularly in ED cases with unusual age onset or atypical symptom presentation [20]. Thus, our patient received MRI diagnostics, which did not reveal pathological findings. Literature describes attention deficits in underweight ED patients, particularly with inefficiencies in set shifting because of rigid and inflexible thinking. Further, despite attention impairment following underweight, patients show excessive detail-focused thinking instead of perceiving the full picture, which could clinically resemble hyperkinetic attention deficit symptoms [21]. Neuropsychological recovery after BMI normalization is described [22,23].

Contrary to these findings, our patient’s pre-existing deficits in attention and self-control were reduced during AN-induced underweight. Third-party interviews and her school reports suggested better performance, contrasting the typically seen intellectual damage in AN [24]. However, FTD-like narrow thinking was present and implies cognitive impairment to some extent, and corresponds with current medical doctrine. It has to be stated that ADHD, in her case re-appearing after recovery from AN, does not impair intellectual functioning, but the access of present cognitive potential. Conversely, superior working memory (WM) performances in AN patients are discussed as appetite-controlling factors [25]. AN-induced higher WM capacity might hence have ‘counteracted’ our patient’s ADHD-impaired WM and improved her working performances. This might explain to some extent a dysfunctional ‘self-help’ for her ADHD-symptoms on a neurocognitive level via developing AN. We do not assume deliberate ‘self-therapy’ of the girl’s ADHD symptoms by developing AN. This is opposed by positive family risk for ED, aetiology of AN with typical anamnesis and present AN-specific ideation, e.g., of ‘most possible slimness’. Yet, it is conceivable that our patient benefitted from various (seemingly) positive individual and socio-psychological consequences of early-stage AN: Her school performance did improve significantly due to the lower impact of ADHD symptoms. As a result of pre-existing frustration following frequent failings or criticism, this might have fuelled her ambition to continue performing. Well-intended praise by caregivers and teachers might have additionally reinforced her in that matter. A better social acceptance and closer integration into peer groups, of which ADHD patients often lack for being perceived as troublemakers, is also a possible factor. Over time of AN development, however, she had increasingly withdrawn from social contacts. At a later stage of AN, the patient suffered from obviously distressful depressive and anxiety symptoms as consequences of underweight, which also resolved with weight gain. Our patient did not ever report thoughts on ADHD and AN being linked. Hence, a deliberate mutual influence is unlikely. The young age of our patient and her psychiatric disorders have to be considered as a limitation of her own reflectivity regarding these complex processes. It is plausible, though, that our patient’s AN-specific behaviour was subconsciously amplified by the aforementioned positive and negative reinforcers.

This report has several limitations: As we can only report one clinical case, the level of evidence is low. Further, observations during the diagnostic and treatment progress were made by a single hospital institution, which might limit objectivity. Yet, standardized diagnostics were applied and therapy progress was evaluated via multi-view sources (the patient herself, caregivers, teachers, healthcare team, therapists), to provide assessments as independent as possible. Further, we accompanied our patient during inpatient and day-care treatment stages, which allowed us a detailed evaluation of symptom changes during her weight increase.

## 4. Conclusions

Our case report shows that AN can have a significant impact on ADHD symptomatology in young patients. AN-induced weight loss might suppress/mask hyperkinetic behaviour, as demonstrated in our case report. Underlying neurobiological explanatory models still have to be investigated. Despite low comorbidity of AN and ADHD among female minors, clinicians should be aware of possible masking effects, particularly in female patients.

## Figures and Tables

**Table 1:** DISYPS-III ratings of ADHD-specific symptoms.

	Self-Report	Caregivers	Therapist Team	Healthcare Team	Teacher of Hospital School
At Inpatient Admission (Under-weight) (PR)	78–89	78–89	24–40	90–96	24–40
After weight-gain (>10th BMI age percentile), no MPH (PR)	/	97–100	97–100	97–100	97–100
After weight-gain (>10th BMI age percentile), with MPH (PR)	78–89	78–89	97–100	97–100	12–23

Notes: DISYPS-III: German diagnostic system for mental disorders according to ICD-10 and DSM-5 for children and adolescents (‘Diagnosesystem für psychische Störungen nach ICD-10 und DSM-5 für Kinder und Jugendliche’); PR: percent range, MPH: methylphenidate, BMI: Body mass index; Interpretation of PR according to DISYPS-III: Marked numbers are to be interpreted as striking values. General interpretation of symptom scales via DISYPS-III: PR 97–100 = particularly striking, PR 90–96 = striking, PR 78–89 = borderline, PR 1–77 = regular findings [15].

## Data Availability

Data sharing is not applicable to this article as no further datasets were analysed during this case report and diagnostical and treatment data are protected by the patient’s and her caregivers’ privacy rights.
